# Poverty, Human Development, and Basic Biology

**DOI:** 10.1371/journal.pbio.0050295

**Published:** 2007-10-23

**Authors:** Liza Gross

## Abstract

Taking a slight departure from our normal fare, PLoS Biology features two articles with a special focus on poverty and human development: one explores the biological mechanisms of health inequalities; the second discusses the value of including local communities in biodiversity conservation.

Nearly half of the world's 6.6 billion people exist on less than US$2 a day [1]. Over 1 billion live in “extreme poverty,” defined by the World Bank as US$1 a day or less. As of 2001, nearly 60% of the poorest people inhabit fragile, vulnerable landscapes—many of which are the highest priorities for biodiversity conservation—and most depend on these natural resources for survival [2]. Yet environmental resources are rapidly deteriorating. Human activity has destroyed biodiversity at an unprecedented rate, at least two to four orders of magnitude above background extinction rates inferred from the fossil record [3]. With global population projected to reach 9 billion by 2050, and 95% of that increase occurring in the developing world [4], poverty and ecosystem health have become increasingly linked.

Today, *PLoS Biology* is publishing two new articles, an Essay and a Community Page, that fall outside the normal scope of our journal. Both address the impacts of growing disparities in social status, but from entirely different perspectives. The Community Page “The Costs of Exclusion: Recognizing a Role for Local Communities in Biodiversity Conservation” (doi:10.1371/journal.pbio.0050289) highlights the consequences of ignoring desperate poverty in the fight to protect the world's most endangered species; the Essay “Biology and Health Inequality” (doi:10.1371/journal.pbio.0050267) explores the health costs of social stratification from a basic biological framework. A primary research journal like *PLoS Biology* does not typically publish articles relating to poverty and human development, but tends to feature the work of basic researchers, who investigate fundamental questions about natural processes to gain knowledge for its own sake—to understand the nature and structure of living systems. Such insights in turn lay the foundation for applied research, which is designed to solve practical, albeit serious, problems. Even articles like Essays and Primers, which do not report new research findings, often highlight efforts to understand fundamental principles or components of biological processes, such as why cetaceans evolved large brains (doi:10.1371/journal.pbio.0050139) or how neurons alter their gene expression in response to their experience (doi:10.1371/journal.pbio.0050055).

We have taken a slight departure from this tradition to participate in the Council of Science Editors' Global Theme Issue on Poverty and Human Development, along with 230 other medical and scientific journals in developed and developing countries, including *PLoS Medicine* and *PLoS Neglected Tropical Diseases*. (View a special collection of the new poverty-related content from these PLoS journals, along with a selected collection of older articles on this theme from all the PLoS journals, at http://collections.plos.org/poverty.php.) The global theme issue was inspired by the 2000 United Nations Millennium Summit (http://www.un.org/millennium/), which outlined an ambitious initiative to eradicate poverty and ensure environmental sustainability. The Council of Science Editors, arguing that achieving the Millennium goals requires the synthesis of scientific knowledge across disciplines, has urged all participating journals to make their poverty theme issues freely available. (As always, all of the PLoS articles are published under our open-access Creative Commons Attribution License: anyone can download, reuse, reprint, modify, distribute, and/or copy any PLoS articles, as long as the original source and authors are properly cited.) While there is no official tally of journals that have agreed to make their content universally available, if all 231 journals comply, they would be contributing to an unprecedented collection of publicly accessible materials (available at http://www.councilscienceeditors.org/globalthemeissue.cfm).

The link between unequal social status and ill health was explored in a study of more than 17,000 civil servants in London nearly 30 years ago. In the landmark Whitehall study, Sir Michael Marmot and his colleagues found a surprising correlation between employment grade and risk of death from cardiovascular disease, with those in the lowest grade experiencing the highest risk of mortality [5]. Subsequent work showed that controlling for conventional coronary risk factors (including smoking, serum cholesterol, and blood pressure) explained only one-third of the social gradient. The biological mechanisms underlying the connection between social status and health have remained obscure, but new hypotheses have emerged from the Whitehall II study, which has followed a second cohort of civil servants for over 20 years. In the new essay “Biology and Health Inequality,” Eric Brunner, who collaborated with Marmot on the second study, describes intriguing parallels in status-related health inequalities between civil servants and nonhuman primate hierarchies and points to evidence suggesting a role for stress-induced neuroendocrine pathways.

Conservation scientists are increasingly finding themselves trying to protect species and ecosystems in places that are inhabited, often by some of the world's poorest people. There is considerable debate about whether species and ecosystem preservation is incompatible with human habitation. In the new article “The Costs of Exclusion: Recognizing a Role for Local Communities in Biodiversity Conservation,” Marc Ancrenaz, Lisa Tabek, and Susan O'Neil describe their efforts to incorporate poverty eradication into two cross-cultural community-based conservation projects: the Kinabatangan Orang-utan Conservation Project in Borneo and the Tree Kangaroo Conservation Program in Papua New Guinea. Ancrenaz and colleagues argue that addressing poverty eradication and biodiversity conservation simultaneously “remains one of our best hopes for achieving tangible and durable results.” In both cases, this strategy has yielded significant conservation gains, including a reduction in nonsustainable timber harvest, fewer wildlife–human conflicts, and a return of wildlife species not seen locally for generations.

Although *PLoS Biology* does not often publish articles that grapple with issues like poverty and human development, we chose to do so here because we believe that the collective output of scientific research can advance the public good. Who knows what connections researchers working in widely disparate disciplines—from evolutionary ecologists to agricultural economists—might make if they had access to the millions of research papers published in the past five years? We applaud the Council of Science Editors' call to make this special collection freely available. Imagine the progress we might see if all the world's scientific literature were truly a public resource.
